# Optimal treatment duration in metastatic renal cell carcinoma patients responding to immune checkpoint inhibitors: should we treat beyond two years?

**DOI:** 10.2340/1651-226X.2025.43876

**Published:** 2025-07-30

**Authors:** Alexander Decruyenaere, Christine Gennigens, Sylvie Rottey, Annouschka Laenen, Emmanuel Seront, Els Everaert, Philip R. Debruyne, Heidi Van Den Bulck, Julie Bastin, Annelies Verbiest, Christof Vulsteke, Peter Schatteman, Daisy Luyten, Sandrine Aspeslagh, Nieves Martinez-Chanza, Marlies De Bock, Thomas Meyskens, Jolanda Verheezen, Barbara Brouwers, Benoit Beuselinck

**Affiliations:** a Department of Medical Oncology, Ghent University Hospital, Gent, Belgium; b Department of Medical Oncology, CHU Liège, Liège, Belgium; c Biostatistics and Statistical Bioinformatics Center, Leuven, Belgium; d Oncologie Médicale, UCL St-Luc, Bruxelles, Belgium; e Medische Oncologie, VITAZ, St Niklaas, Belgium; f Kortrijk Cancer Centre, General Hospital AZ Groeninge, Kortrijk, Belgium; g Medical Technology Research Centre (MTRC), School of Allied Health and Social Care, Anglia Ruskin University, Chelmsford, UK; h School of Nursing and Midwifery, University of Plymouth, Plymouth, UK; i Medische Oncologie, AZ Imelda, Bonheiden, Belgium; j Medische Oncologie, Heilig Hart ziekenhuis, Lier, Belgium; k Department of Oncology, Multidisciplinary Oncological Center Antwerp, Antwerp University Hospital, Edegem, Belgium; l Center for Oncological Research (CORE), Antwerp University, Antwerpen, Belgium; m Medische Oncologie, Maria Middelares Ziekenhuis, Gent, Belgium; n Uro Onco Unit, Urology, AZORG, Aalst, Belgium; o Medische Oncologie, Jessa Ziekenhuis, Hasselt, Belgium; p Medische Oncologie, UZBrussel, Brussel, Belgium; q Department of Medical Oncology, Institut Jules Bordet – Hôpital Universitaire de Bruxelles, Université Libre de Bruxelles (ULB), Brussels, Belgium; r Medische Oncologie, AZ Delta, Roeselare, Belgium; s Medische Oncologie, Klina, Brasschaat, Belgium; t Medische Oncologie, Trudo Ziekenhuis, St Truiden, Belgium; u Medische Oncologie, St Jan Ziekenhuis, Brugge, Belgium; v General Medical Oncology, University Hospital Leuven, Leuven, Belgium

**Keywords:** Renal cell carcinoma, immune checkpoint inhibitors, optimal treatment duration, treatment discontinuation

## Abstract

**Background and purpose:**

Optimal treatment duration is unknown in metastatic renal cell carcinoma (mRCC) responding to immune checkpoint inhibitors (ICPIs). Prolonged treatment can lead to late toxicity, burden for day clinics and financial impact.

**Patients and methods:**

This multicenter retrospective study included mRCC patients responding to ipilimumab/nivolumab in first-line or nivolumab in later lines, who were treated for at least 21 months and did not stop for toxicity. Progression-free survival (PFS), overall survival (OS), and cancer-specific survival (CSS) were modeled non- and semi-parametrically. The effect of elective ICPI discontinuation (i.e. treatment interruption at the clinician’s discretion) between 21 and 25 months on PFS was assessed by a causal inference approach using artificial censoring along with inverse probability of censoring weighting.

**Results:**

Ninety-five patients were included with a median follow-up of 62.1 (95% confidence interval [CI]: 57.3–67.5) months. Fifty-four received ipilimumab/nivolumab, whereas 41 patients received nivolumab, for a median treatment duration of 33.8 (95% CI: 28.5–39.6) months.

Fifty-seven patients discontinued ICPIs electively. Three-year PFS after discontinuation was 57.1% (95% CI: 34.3–95.1), 3-year OS 67.5% (95% CI: 37.0–100.0), and 3-year CSS 90.0% (95% CI: 73.2–100.0).

Fifteen (15.8%) patients discontinued ICPIs between 21 and 25 months. Compared to 80 patients who were treated longer, they had more often a metachronous metastatic pattern (*p* = 0.048) and a complete response (*p* = 0.045). Elective ICPI stop between 21 and 25 months did not significantly impact the hazard for progression/death (adjusted HR 1.08, 95% CI: 0.64–1.84, *p* = 0.766).

**Interpretation:**

Among mRCC patients responding to ICPI, elective therapy discontinuation approximately 24 months after initiation does not appear to compromise outcomes compared to continuing therapy.

## Optimal treatment duration in metastatic renal cell carcinoma patients responding to immune checkpoint inhibitors: should we treat beyond two years?

**Figure:**
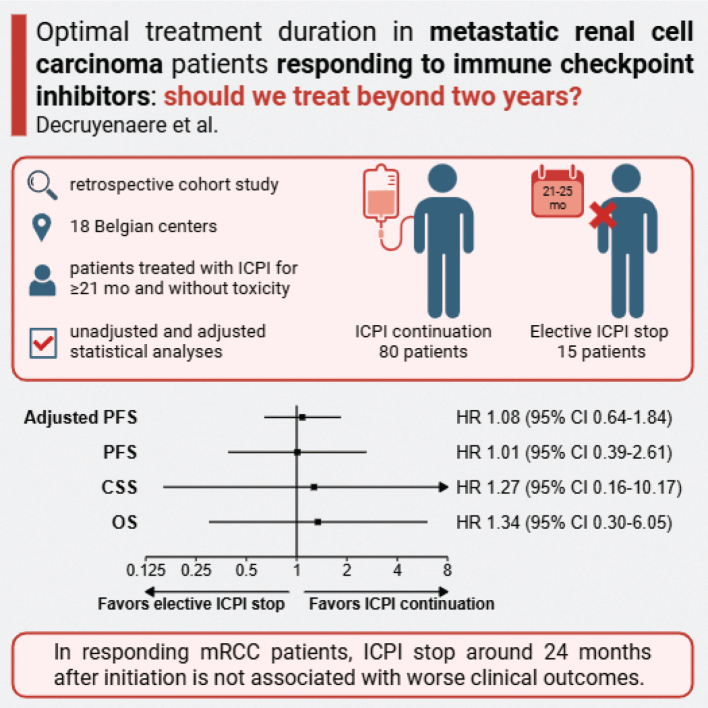

## Background

Immune checkpoint inhibitors (ICPIs) are routinely used in the management of metastatic renal cell carcinoma (mRCC), either as second- or further-line treatment (nivolumab) or as first-line treatment (ipilimumab/nivolumab). In case of partial or complete response (PR/CR), durable disease control may be observed, even after ICPI discontinuation for toxicity or at the physician/patient’s decision [1, 2].

The persisting responses after ICPI discontinuation raise the question how long these patients should be treated. Prolonged treatment can lead to late toxicities, burden for day clinics and financial impact. Unlike in other tumor types, where treatment duration in pivotal studies was limited to 24 months, nivolumab or ipilimumab/nivolumab administration was not restricted in time in RCC trials [3]. Hence, there are no international or national guidelines on ICPI discontinuation in mRCC patients.

Retrospective studies consistently reported ‘favorable outcome’ in terms of progression-free survival (PFS) and/or overall survival (OS) after ICPI discontinuation for PR/CR and/or toxicities after 12–24 months of treatment, mostly in melanoma and non-small cell lung carcinoma (NSCLC). Depth of response is often associated with PFS and/or OS. Hence, limiting ICPI administration to 24 months in responding patients seems a reasonable option.

Only five studies examined the relationship between ICPI duration of exposure and outcome. One prospective study, randomizing NSCLC patients between a fixed treatment duration of 12 months and a treatment duration > 12 months, showed inferior outcome with the former [4]. A retrospective study in NSCLC patients showed improved 1-year PFS and OS post-discontinuation after 24 months of treatment compared to patients who were treated for 6–24 months, with the poorest PFS observed in patients treated between 6 and 11 months [5]. However, another retrospective study in NSCLC patients showed similar OS in patients treated for 24 or > 24 months [6]. In melanoma patients achieving CR, a treatment duration < 6 months was associated with worse OS [7]. Finally, in patients with urothelial carcinoma (UCC), a trend for improved PFS was observed in patients treated with ICPI > 12 months [8]. Hence, four of these five studies show that longer ICPI exposure is correlated with improved outcome.

Data on the relationship between ICPI exposure and outcome in mRCC are currently lacking. We aimed to describe outcome of mRCC patients after they discontinued ICPIs for PR/CR and to determine whether ICPIs can be electively discontinued after approximately 24 months of treatment without compromising these outcomes.

## Materials and methods

This multicenter, retrospective dynamic cohort study included mRCC patients of all histologic subtypes treated with ipilimumab/nivolumab in first-line or nivolumab in later lines who reached PR or CR per iRECIST [9], were treated for at least 21 months, and did not experience treatment-limiting toxicity. Cases were collected from 18 Belgian academic and general hospitals, without restriction to a specific time period. Elective ICPI stop was defined as ICPI discontinuation at the discretion of the physician and/or patient in absence of toxicity, disease progression, and death. Mild toxicities could be present as long as they did not oblige clinicians to discontinue therapy. Patients who needed to stop for severe toxicities were excluded. In the absence of guidelines and experimental data, the treating physician can decide for treatment discontinuation based on the depth and duration of response and based on results after ICPI discontinuation in other tumor types. This study was approved by the central ethics committee of UZLeuven (S67880) and the local ethics committees of the participating hospitals. The need for informed consent was waived due to the retrospective nature of this study.

The first objective was to study the further evolution of response after therapy stop in patients who discontinued ICPIs for PR/CR at any moment. The second objective was to study the added value of continuing treatment with ICPI after 24 months in patients with PR/CR. Since nobody stopped exactly at 24 months, discontinuation within a grace period between 21 and 25 months was allowed. Sensitivity analyses for other grace periods were conducted. Patients with stable disease (SD) as best response on ICPIs were excluded from the analysis, because patients with SD as best response rarely display long lasting responses and in these patients, the clinician will be less prone to consider ICPI discontinuation. Hence, the number of patients with ICPI discontinuation after SD as best response would be too low in order to draw reliable conclusions.

PFS described the time to disease progression or death from any cause (whichever occurred first). OS measured the time to death from any cause. Cancer-specific survival (CSS) represented the time to cancer-related death. Central radiologic review was not foreseen in the study. iRECIST calculation was performed by the treating oncologist, reflecting routine practice in Belgium.

Continuous and categorical baseline characteristics were compared between groups using the Wilcoxon-Mann-Whitney test and Chi-squared test (or Fisher’s exact test in case of low expected cell count), respectively. Survival functions for follow-up time, treatment duration, PFS and OS were calculated non-parametrically using the Kaplan-Meier estimator. Hazards for PFS and OS were modeled semi-parametrically by a Cox model and compared between patients who continued vs. electively discontinued their ICPIs between 21 and 25 months. The sub-distribution hazard for CSS was modeled by a Fine-Gray model and compared likewise, treating non-cancer-related death as a competing event. Subgroup analyses were performed by adding the interaction between elective ICPI stop indicator and subgroup (stratified analysis in case of non-proportional hazards).

However, these unadjusted analyses may be prone to bias, which compromises any causal interpretation and does not address the question whether the clinical outcomes would have changed if all patients had continued vs. electively discontinued their ICPIs between 21 and 25 months:

1. Immortal time bias: patients who electively discontinued their ICPIs were not at risk for disease progression or death between the landmark time of 21 months and their time of elective stop within 21–25 months (by design), whereas patients who always continued their ICPIs were already at risk following the landmark time, which may have introduced an artificial survival benefit for the former.
2. Confounding bias: the risk factor profile may vary (at baseline and/or over time) between those who continued vs. electively discontinued their ICPIs.
3. Misclassification bias: patients receiving ongoing ICPIs who were censored before the cut-off of 25 months were classified in the group of ICPI continuation (beyond 25 months), while they still could have electively discontinued their ICPIs before 25 months.

Overcoming these biases is non-trivial as traditional adjustment approaches fall short [10, 11]. Therefore, we followed the approach outlined in Hernán et al. [12] to compare dynamic treatment regimens by artificially censoring patients and then adjusting for selection bias due to this censoring by inverse probability of censoring weighting (IPCW). Specifically, we considered the effect of two treatment regimens on PFS in all patients: always continue ICPIs (until progression/death) vs. electively discontinue ICPIs during the grace period of 21–25 months. As soon as patients deviated from a regime (for instance, elective ICPI stop in the former and ICPI continuation beyond 25 months in the latter), they were censored in that particular regime arm. Patients complying with a regime were followed until progression/death. As this treatment censoring may depend on baseline and time-varying confounders, weights (unstabilized and truncated at the 1st and 99th percentiles) were calculated via IPCW to correct for this dependent censoring and subsequently applied to a weighted Cox marginal structural model that described the relationship between treatment regime and PFS. As such, this approach may yield the causal effect of elective ICPI stop between 21 and 25 months vs. ICPI continuation on PFS in a pseudo-population in which everybody followed one of these two regimes. Sensitivity analyses for other treatment regimens (using different grace periods during which patients were allowed to electively discontinue their ICPIs) were conducted. Adjusted analyses of OS/CSS were not performed due to the low number of deaths.

Following baseline confounders were considered: age (< 65 years vs. ≥ 65 years), sex (male vs. female), Eastern Cooperative Oncology Group performance status (ECOG-PS) (0 vs. 1–3), International Metastatic RCC Database Consortium (IMDC) risk category (good vs. intermediate/poor), metastatic pattern (synchronous vs. metachronous), number of metastatic sites (1 vs. ≥ 2), histology (clear cell vs. non-clear cell), sarcomatoid features (no vs. yes), ICPI type (nivolumab vs. ipilimumab/nivolumab), and best objective response (partial vs. complete). Nephrectomy (no vs. yes) was used as a time-dependent confounder. Statistical analysis was performed in R version 4.2.2 [10].

## Results

### Baseline characteristics

The study cohort comprised 95 mRCC patients (characteristics at start of ICPI in Table 1). Forty-one (43.2%) patients received nivolumab, whereas 54 (56.8%) received ipilimumab/nivolumab, for a median treatment duration of 33.8 (95% confidence interval [CI] 28.5–39.6) months (Supplemental Figure 1). Fifty-four (56.8%) patients had a PR and 41 (43.2%) had a CR, achieving a median tumor shrinkage of -82.0% (range -31.0 to -100.0; Supplemental Figure 2).

**Table 1:** Baseline characteristics at start of ICPI therapy (overall and by elective ICPI stop).

Characteristic	All patients (*n* = 95)	No elective stop 21–25 months (*n* = 80)	Elective stop 21–25 months (*n* = 15)	*p*-value†
Age				
Median (range) – years	66.0 (35.7–88.9)	65.6 (35.7–87.3)	67.8 (48.4–88.9)	0.268
Distribution – no. (%)				0.355
< 65 years	42 (44.2)	37 (46.3)	5 (33.3)	
≥ 65 year	53 (55.8)	43 (53.8)	10 (66.7)	
Sex – no. (%)				0.544
Male	68 (71.6)	56 (70.0)	12 (80.0)	
Female	27 (28.4)	24 (30.0)	3 (20.0)	
ECOG performance status – no. (%)				0.691
0	53 (55.8)	46 (57.5)	7 (46.7)	
1	37 (38.9)	29 (36.3)	8 (53.3)	
2	3 (3.2)	3 (3.8)	0 (0.0)	
3	2 (2.1)	2 (2.5)	0 (0.0)	
IMDC risk category – no. (%)				0.321
Good	12 (12.6)	12 (15.0)	0 (0.0)	
Intermediate	65 (68.4)	53 (66.3)	12 (80.0)	
Poor	18 (18.9)	15 (18.8)	3 (20.0)	
Metastatic pattern – no. (%)				0.048
Synchronous	41 (43.2)	38 (47.5)	3 (20.0)	
Metachronous	54 (56.8)	42 (52.5)	12 (80.0)	
No. metastatic sites – median (range)	2 (1–7)	2 (1–7)	2 (1–4)	0.314
Lymph nodes – no. (%)	47/95 (49)	40/80 (50)	7/15 (47)	
Lung – no. (%)	60/95 (63)	49/80 (61)	11/15 (73)	
Liver – no. (%)	19/95 (20)	16/80 (20)	3/15 (20)	
Brain – no. (%)	3/95 (3)	3/80 (4)	0/15 (0)	
Pancreas – no. (%)	8/95 (8)	7/80 (9)	1/15 (7)	
Bone – no. (%)	33/95 (35%)	30/80 (38)	3/15 (20)	
Histology – no. (%)				1.00
Clear cell	88 (92.6)	74 (92.5)	14 (93.3)	
Non-clear cell	7 (7.4)	6 (7.5)	1 (6.7)	
Sarcomatoid features – no. (%)				1.00
No	66 (69.5)	55 (68.8)	11 (73.3)	
Yes	29 (30.5)	25 (31.3)	4 (26.7)	
ICPI type – no. (%)				0.403
Nivolumab	41 (43.2)	36 (45.0)	5 (33.3)	
Ipilimumab/nivolumab	54 (56.8)	44 (55.0)	10 (66.7)	
Previous VEGFR-TKI or mTOR-inhibitor				
One line	30/95 (32)	28/80 (35)	2/15 (13)	
Two lines	5/95 (5)	3/80 (4)	2/15 (13)	
Three lines	1/95 (1)	1/80 (1)	0/15 (0)	
Four lines	3/95(3)	3/80 (4)	0/15 (0)	
Best objective response				
Median (range) – % decrease in SLD	82.0 (31.0–100.0)	79.5 (31.0–100.0)	100.0 (32.0–100.0)	0.049
Distribution – no. (%)				0.045
Partial	54 (56.8)	49 (61.3)	5 (33.3)	
Complete	41 (43.2)	31 (38.8)	10 (66.7)	
Nephrectomy – no. (%)				1.00
No*	18 (18.9)	15 (18.8)	3 (20.0)	
Yes	77 (81.1)	65 (81.3)	12 (80.0)	

ECOG: Eastern Cooperative Oncology Group; ICPI: immune checkpoint inhibitor; IMDC: International Metastatic RCC Database Consortium; SLD: sum of longest diameter; VEGFR-TKI: vascular endothelial growth factor receptor tyrosine kinase inhibitor; mTOR: mammalian target of rapamycin.

* 7 patients had a deferred nephrectomy after baseline.

† Continuous and categorical variables were compared between groups using the Wilcoxon-Mann–Whitney test and Chi-squared test (or Fisher’s exact test in case of low expected cell count), respectively.

**Figure 1:**
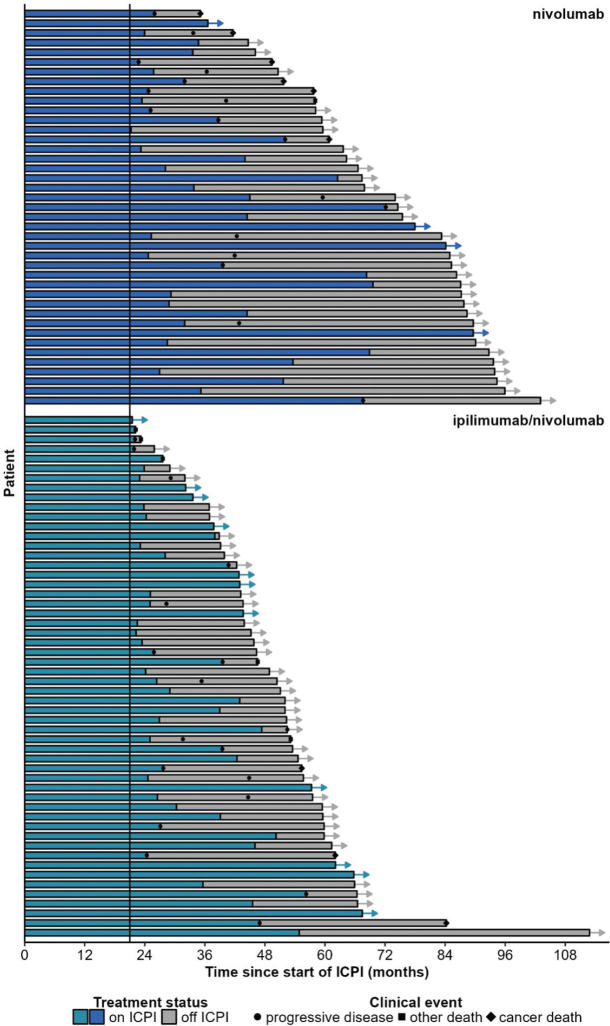
Swimmer plot by type of ICPI.

After median follow-up of 62.1 (95% CI: 57.3–67.5) months, 35 (36.8%) cases of disease progression, nine (9.5%) cancer-related deaths, and six (6.3%) non-cancer-related deaths occurred. A swimmer plot depicting these events is presented by ICPI type in Figure 1 and by best objective response in Supplemental Figure 3. Median PFS (mPFS), median OS (mOS), and median CSS (mCSS) were not reached (NR). Five-year survival rates from ICPI initiation were 57.7% (95% CI: 47.6–69.9) for PFS, 82.9% (95% CI: 74.3–92.5) for OS, and 91.0% (95% CI: 84.4–98.3) for CSS (Supplemental Figures 4–6).

The univariate association between baseline characteristics and clinical outcomes is plotted in Supplemental Figure 7. CR was associated with PFS (*p* < 0.001), OS (*p* < 0.001), and CSS (*p* = 0.003). ICPI type and IMDC risk category were not significantly associated with clinical outcomes. The corresponding survival functions are shown in Supplemental Figure 8.

### ICPI (dis)continuation pattern

Twenty-three (24.2%) patients continued ICPIs (median 27.7 months, 95% CI: 25.8–40.7) until progression/death, while 15 (15.8%) patients were still on ICPIs (median 43.7 months 95% CI: 37.8–78.0) in absence of progression/death. Fourteen (14.7%) patients electively discontinued ICPIs at any time (median 25.2 months, 95% CI: 24.7–45.0) and experienced progression/death later on. Finally, 43 (45.3%) patients electively discontinued ICPIs at any time (median 33.8 months, 95% CI: 28.5–43.0) and did not yet experience progression/death during follow-up.

**Figure 2:**
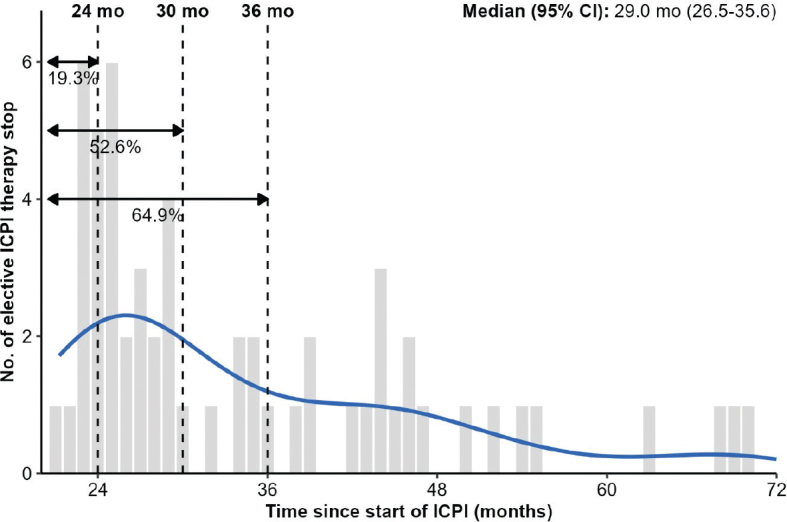
Distribution of stopping times in patients electively discontinuing their ICPI treatment.

The distribution of stopping time in the 57 (60.0%) patients electively discontinuing their ICPIs is shown in Figure 2, peaking around 24 months after ICPI initiation and steadily decreasing thereafter with median stopping time of 29.0 (95% CI: 26.5–35.6) months. The corresponding cumulative incidence of elective ICPI stop is presented in Supplemental Figure 9. Fifteen (26.3%) of them discontinued between 21 and 25 months (baseline characteristics in Table 1). These patients had significantly more often a metachronous metastatic pattern (*p* = 0.048) and a CR (*p* = 0.045) compared to patients who did not (yet) electively discontinue their treatment within this period.

One-year, 2-year, and 3-year PFS after discontinuation was 85.7% (95% CI: 69.2–100.0), 57.1% (95% CI: 34.3–95.1), and 57.1% (95% CI: 34.3–95.1), respectively. One-year, 2-year, and 3-year OS after discontinuation was 100.0% (95% CI: 100.0–100.0), 90.0% (95% CI: 73.2–100.0), and 67.5% (95% CI: 37.0–100.0), respectively. One-year, 2-year, and 3-year CSS after discontinuation was 100.0% (95% CI: 100.0–100.0), 90.0% (95% CI: 73.2–100.0), and 90.0% (95% CI: 73.2–100.0), respectively (Supplemental Figure 10).

### Impact of elective ICPI stop between 21 and 25 months on PFS

**Figure 3:**
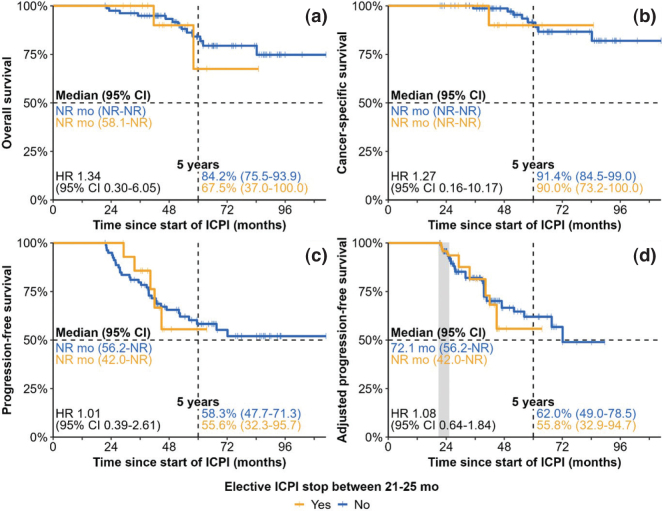
Survival outcomes of ICPI continuation vs. elective ICPI stop between 21 and 25 months. Analyses of overall survival (A), cancer-specific survival (B), and progression-free survival (C) are unadjusted, while adjusted progression-free survival (D) follows the causal approach outlined in the Methods section. The grace period is indicated by the grey segment.

The association between elective ICPI stop between 21 and 25 months and clinical outcomes is shown in Figure 3A–C. It was not significantly associated with PFS (HR 1.01, 95% CI: 0.39–2.61, *p* = 0.989), OS (HR 1.34, 95% CI: 0.30–6.05, *p* = 0.699), or CSS (HR 1.27, 95% CI: 0.16–10.17, *p* = 0.822). mPFS, mOS, and mCSS were not reached in patients continuing ICPI and in patients electively stopping ICPI between 21 and 25 months. There was no (statistically significant) differential association between elective ICPI stop between 21 and 25 months and PFS in subgroup analyses (Supplemental Figure 11).

**Figure 4:**
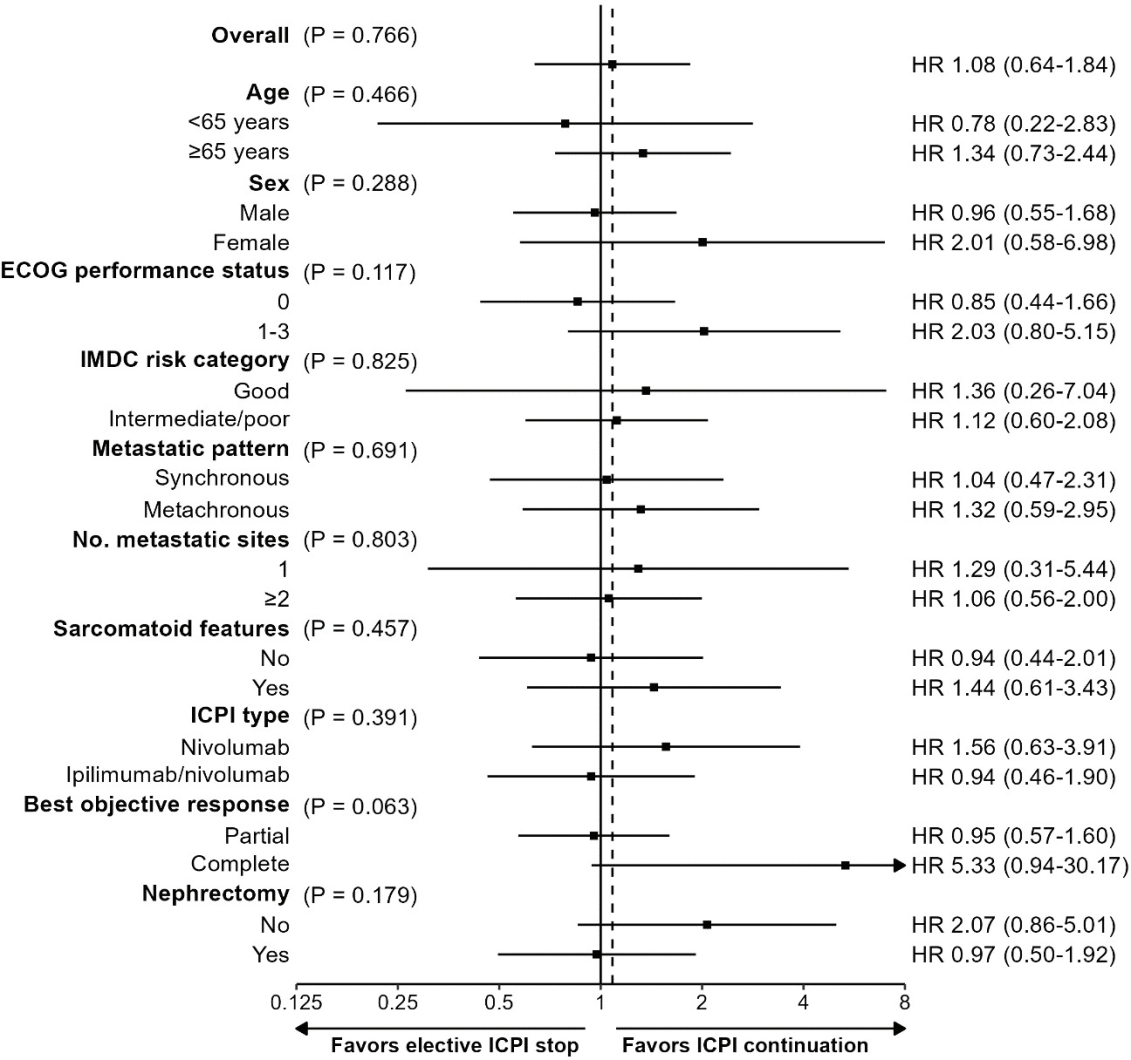
Forest plot of adjusted progression-free survival for ICPI continuation vs. elective ICPI stop between 21 and 25 months by subgroup. Interaction for histology could not be fitted due to sparse data.

To mitigate the biases inherent to a naive analysis, we followed the causal approach outlined in the Methods section. This was performed for PFS, but not for OS or CSS because of the low number of observed deaths. The adjusted PFS analysis indicate that the hazard for progression/death would not have significantly increased (HR 1.08, 95% CI: 0.64–1.84, *p* = 0.766, Figure 3D) if all patients would have electively stopped between 21 and 25 months (mPFS NR, 95% CI: 42.0-NR months) vs. if all patients would have continued their ICPI treatment (mPFS 72.1, 95% CI: 56.2-NR months). In addition, there did not seem to be a (statistically significant) differential treatment effect between elective ICPI stop and PFS across the subgroups considered in Figure 4, although these results should be interpreted with caution due to small patient and event numbers. Continuing ICPIs only yielded a 5-year PFS rate benefit of 6.2% (95% CI: -26.7 to 39.1) and a 5-year mean PFS time benefit of 1.0 (95% CI: -6.4 to 8.5) months, despite an average additional exposure to ICPI of 26.4 (95% CI: 23.1 to 29.6) months at 5 years.

Sensitivity analyses for other grace periods (during which patients were allowed to electively discontinue their ICPI treatment) are presented in Supplemental Table 1, indicating that elective ICPI stop within these periods did not have a statistically significant effect on PFS either.

## Discussion

ICPIs represent a cornerstone of the management of mRCC. They are usually discontinued in case of disease progression or serious adverse events. However, for some (especially responding) patients, discontinuation may be optional, as ICPIs may induce a so-called legacy effect that describes a persisting response even after treatment cessation.

In our study, patients with mRCC achieving PR/CR to ipilimumab/nivolumab or nivolumab have favorable long-term survival outcomes, as reflected by a 5-year PFS rate of 57.7% (95% CI: 47.6–69.9) and 5-year CSS rate of 91.0% (95% CI: 84.4–98.3) since initiation of ICPIs. Deeper responses (CR vs. PR) were especially associated with prolonged survival, highlighting the prognostic value of depth of response. IMDC risk category was neither associated with survival outcomes nor was ICPI type, suggesting that the prognostically unfavorable value of IMDC intermediate and poor risk tumors was compensated by ICPI efficacy.

Using a causal approach that accounted for immortal time bias and confounding bias, including depth of response, we found that elective ICPI stop between 21 and 25 months did not significantly impact the hazard for progression/death. In particular, 5-year PFS would only be 6.2% lower in absolute terms, translating to an estimated number needed to treat of 17 in favor of continuing ICPI treatment, while potentially saving, on average, 26.4 months of additional therapy per patient. Moreover, none of the subgroups seemed to benefit from continuing ICPI treatment. Sensitivity analyses using grace periods other than 21–25 months did not change the interpretation.

The prevalence of sarcomatoid dedifferentiation was relatively high in this series (30%), reflecting the fact that ICPIs are particularly efficient in RCCs with sarcomatoid dedifferentiation.

Many studies report on the globally favorable outcome after ICPI-discontinuation for PR/CR or toxicity in several tumor types. Supplemental Table 2A summarizes the data of 10 studies in melanoma [7, 13–21] and Supplemental Table 2B in other tumor types [5, 6, 8, 22–27]. All studies report favorable PFS/OS after treatment discontinuation. Studies that looked at the impact of depth of response (CR vs. PR) on outcome consistently report better outcome after CR. In melanoma, CR is a valid surrogate marker for long-term survival in patients treated with ICPI, while CR as a sign for treatment cessation has not been widely adopted in advanced NSCLC due to low CR rates (< 5%). The ESMO consensus on melanoma suggests to discontinue treatment if two CT scans show CR after at least 6 months of therapy and to discontinue treatment in case of PR after 2 years of immune therapy [28].

One meta-analysis of 16 prospective mRCC studies examined treatment-free survival (TFS, as calculated from therapy cessation) in objective responders who discontinued ICPIs. Of the 572 responders (PR/CR), 327 stopped ICPIs. Pooled TFS rates were 57% at 6 and 50% at 12 months for patients treated with dual ICPIs and 30% at 6 and 21% at 12 months for patients treated with ICPI monotherapy [2].

In a meta-analysis of 36 studies with 2,180 patients with metastatic tumors who discontinued ICPIs for reasons other than progressive disease (PD), mPFS from the date of treatment discontinuation was 24.7 months and PFS-rate at 1, 2, and 3 years 69.8%, 51.0% and 34.0%, respectively. mPFS was significantly longer for patients with melanoma, as compared with NSCLC and RCC, and for patients treated with dual ICPIs as compared to monotherapy [29].

Four retrospective and one prospective studies report on impact of duration of ICPI-therapy (Supplemental Table 2C). Checkmate153 is the only prospective de-escalation trial randomizing NSCLC patients after 1 year of nivolumab between stop or continuing nivolumab. Continuing nivolumab > 1 year improved OS in patients reaching PR/CR, but not in patients reaching only SD [4]. In a study on 139 NSCLC patients who discontinued without disease progression, but for immune-related adverse events (60%) or other causes (40%) at different time moments, 96 patients completed 24 months of ICPI and then discontinued, while 43 patients were treated between 12 and 23 months. One-year PFS rate was 81% versus 71%. One-year OS rate was 96% versus 90%. mPFS was 20.7 months in patients treated between 6 and 11 months and not reached in patients treated between 12 and 17 months, between 18 and 23 months and > 24 months [5]. In 706 advanced NSCLC patients receiving first-line immunotherapy and still on treatment at 24 months, immunotherapy was stopped after 24 months in 113 patients and continued > 24 months in 593 patients, following physician’s choice. Two-year OS from 24 months was similar: 79% in the fixed-duration and 81% in the indefinite-duration group. However, response data are not available and response might have been more important in patients who stopped at 24 months. Baseline patients’ characteristics of both subgroups such as smoking status, PDL1 positivity, histologic subtype and practice setting were in favor of the fixed duration group [6]. In the absence of progression or treatment-limiting toxicity, 117 advanced melanoma patients with CR discontinued anti-PD1-therapy. In patients treated for > 6 months, the risk of relapse after treatment discontinuation was low, while patients who were treated for < 6 months had a significant higher risk of progression. mPFS was not reached versus 18.9 months (*p* < 0.05). There was no difference in risk of progression between patients treated for 6–12, 12–18, 18–24 or > 24 months [7]. After a median treatment duration of 6.9 months, 83 platinum-refractory metastatic UCC patients reaching PR/CR, discontinued pembrolizumab before progression. Propensity score-matched landmark analysis revealed no significant OS difference between patients who continued or discontinued pembrolizumab at 6, 12, and 18 months (*p* = 0.91, 0.99, and 0.25, respectively), however, a trend was noted for better PFS if the treatment was longer than 1 year compared to shorter than 1 year (*p* = 0.079) [8]. In summary, four out of these five studies indicate a positive correlation between treatment duration and outcome. Shorter ICPI exposure, especially below 6–12 months, seems correlated to poorer outcome. None of these studies looked at discontinuation cut-offs beyond 24 months.

Strengths of our study include the multicenter, homogeneous study cohort of patients who all have reached PR/CR and did not stop for toxicity. We also performed a rigorous adjustment approach to tackle the biases inherent to addressing the causal question whether ICPIs could be electively stopped.

Nevertheless, caution is warranted in the interpretation of the study results due to some methodological limitations. Firstly, ECOG-PS was actually time-dependent, but information over time was lacking. Objective response may also have varied over time, but in all cases, best objective response was achieved prior to the landmark time of 21 months (as such, it can be considered a baseline and not a time-dependent variable). In addition, the adjustment approach assumed that all confounders were measured, whereas some patients may have electively discontinued treatment based on prognostic factors that have not been measured. This may leave residual confounding bias in the estimation of the effect of elective ICPI stop, thereby compromising a causal interpretation. We would expect residual confounding bias in favor of discontinuing patients, as they were probably better in terms of prognosis compared to continuing patients, so proper adjustment would lead to worse survival for the former and better survival for the latter. For instance, patients whose tumor lesions were again increasing after achieving best response, were presumably more likely to continue with ICPIs until progressive disease was formally confirmed. Failing to adjust for sum of longest diameter over time may therefore induce an artificial survival disadvantage for ICPI continuation compared to elective ICPI stop and underestimate any harmful effect (or overestimate any protective effect) of elective ICPI stop. Moreover, by protocol, our study excluded patients with SD as best response. Hence, the question of optimal treatment duration in these patients was not answered. Other limitations included the small number of patients in the elective discontinuation group between 21 and 25 months, leading to wide confidence intervals around the effect estimates (even more pronounced in the subgroup analyses, where some subgroup-specific effects could not be reliably estimated for all baseline characteristics), and the absence of central radiologic review. Finally, we did not perform an adjusted analysis for OS or CSS due to the low number of observed deaths. We plan to update these study results after additional years of follow-up. Prospective randomized studies are nonetheless warranted.

In summary, among mRCC patients responding to ICPI, elective therapy discontinuation approximately 24 months after initiation does not appear to compromise survival outcomes compared to continuing therapy. This study was, however, not powered to detect modest survival differences.

## Supplementary Material



## Data Availability

Data used in this article are available upon reasonable request.
