# Supplementary material for: Optimal treatment duration in metastatic renal cell carcinoma patients responding to immune checkpoint inhibitors: should we treat beyond two years?
Source: Acta Oncol. 2025 Jul 30;64:43876. doi: 10.2340/1651-226X.2025.43876 (PMC12320143; doi:10.2340/1651-226X.2025.43876)

Supplementary material has been published as submitted. It has not been copyedited, or typeset by Acta Oncologica

Supplemental Figure 1. ICPI treatment duration. Abbreviations: CI, confidence interval; ICPI, immune checkpoint inhibitor; mo, month.

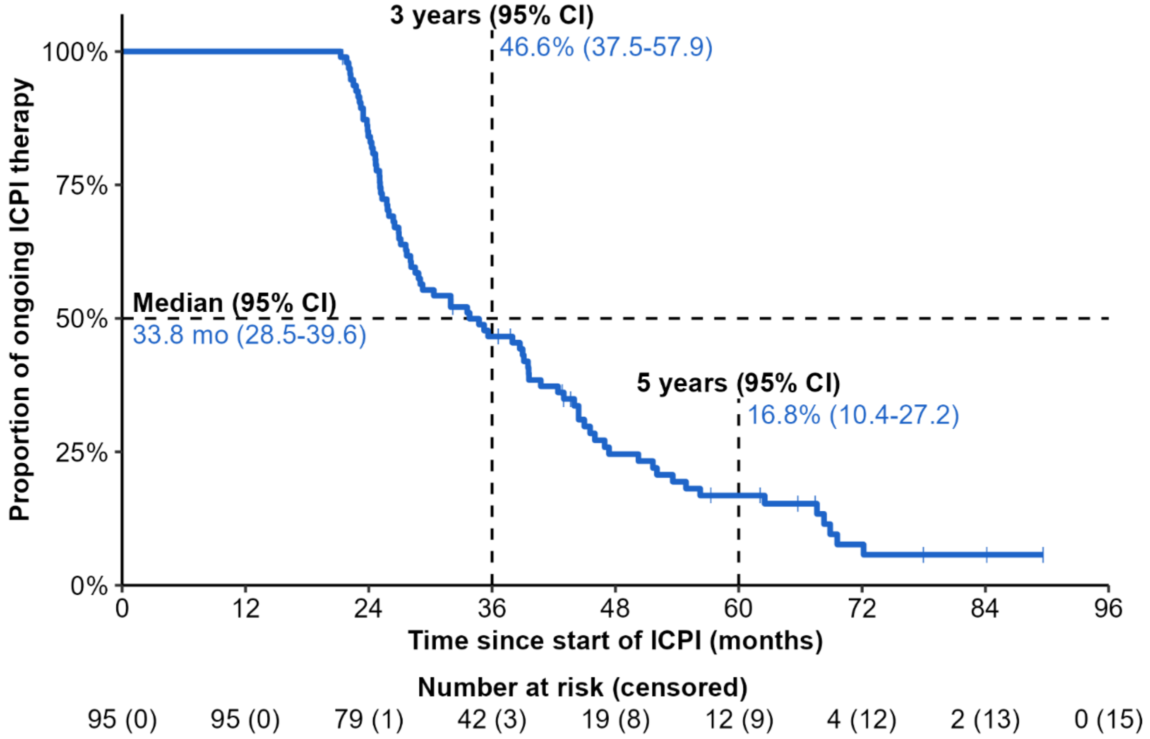

Supplemental Figure 2. Waterfall plot by type of ICPI. Abbreviations: CI, confidence interval; ICPI, immune checkpoint inhibitor.

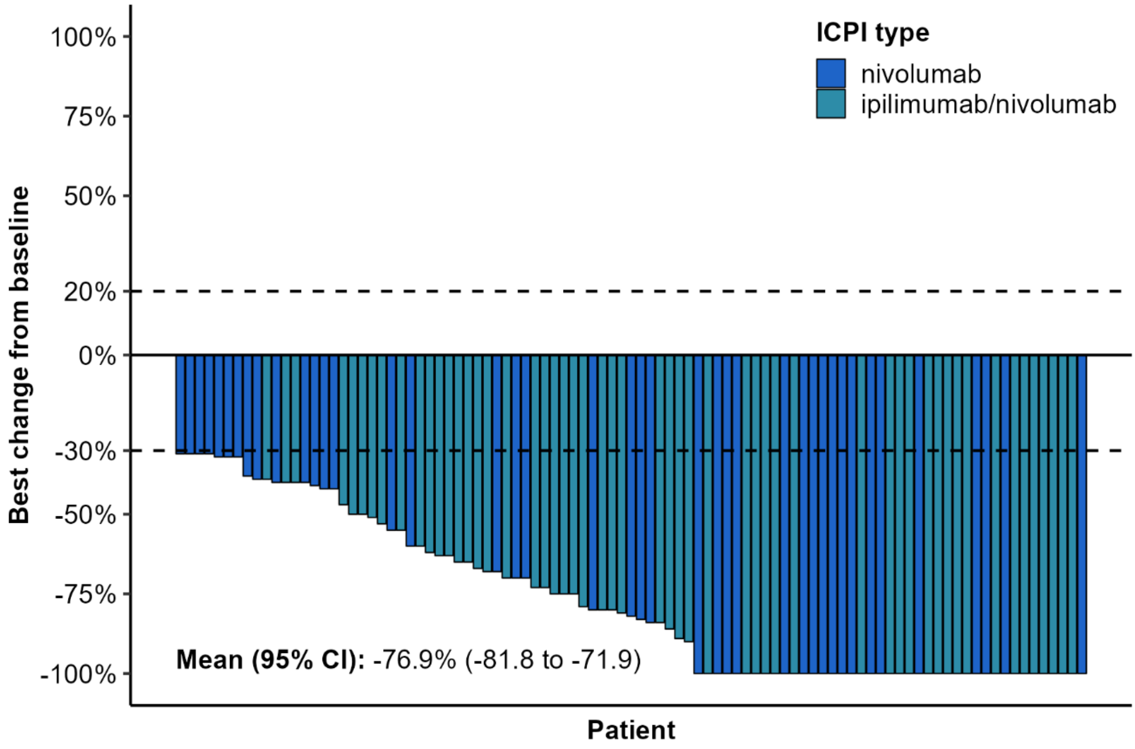

Supplemental Figure 3. Swimmer plot by best objective response. Abbreviations: CR, complete response; ICPI, immune checkpoint inhibitor; PR, partial response.

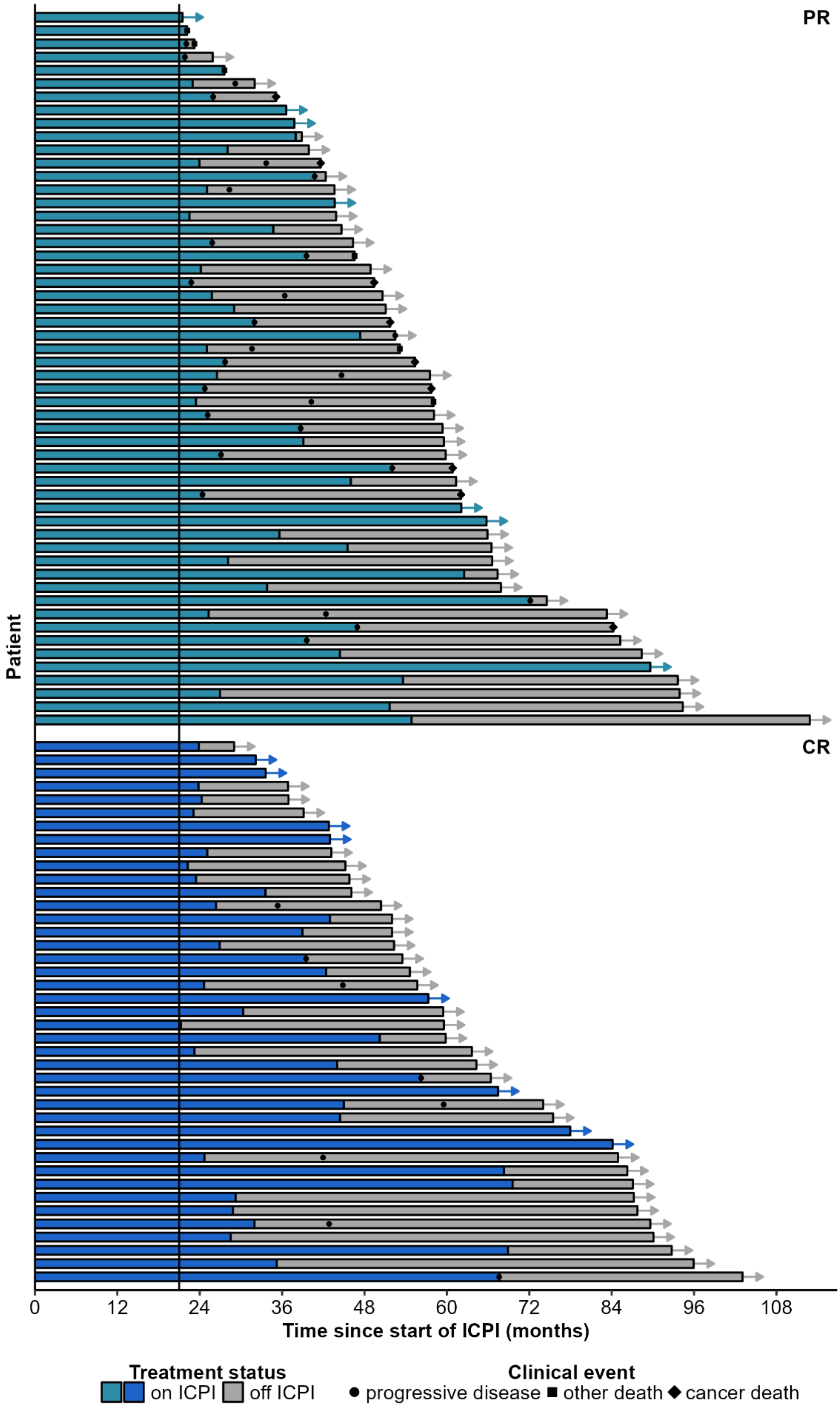

Supplemental Figure 4. Progression-free survival in the overall population. Abbreviations: CI, confidence interval; ICPI, immune checkpoint inhibitor; mo, month; NR, not reached.

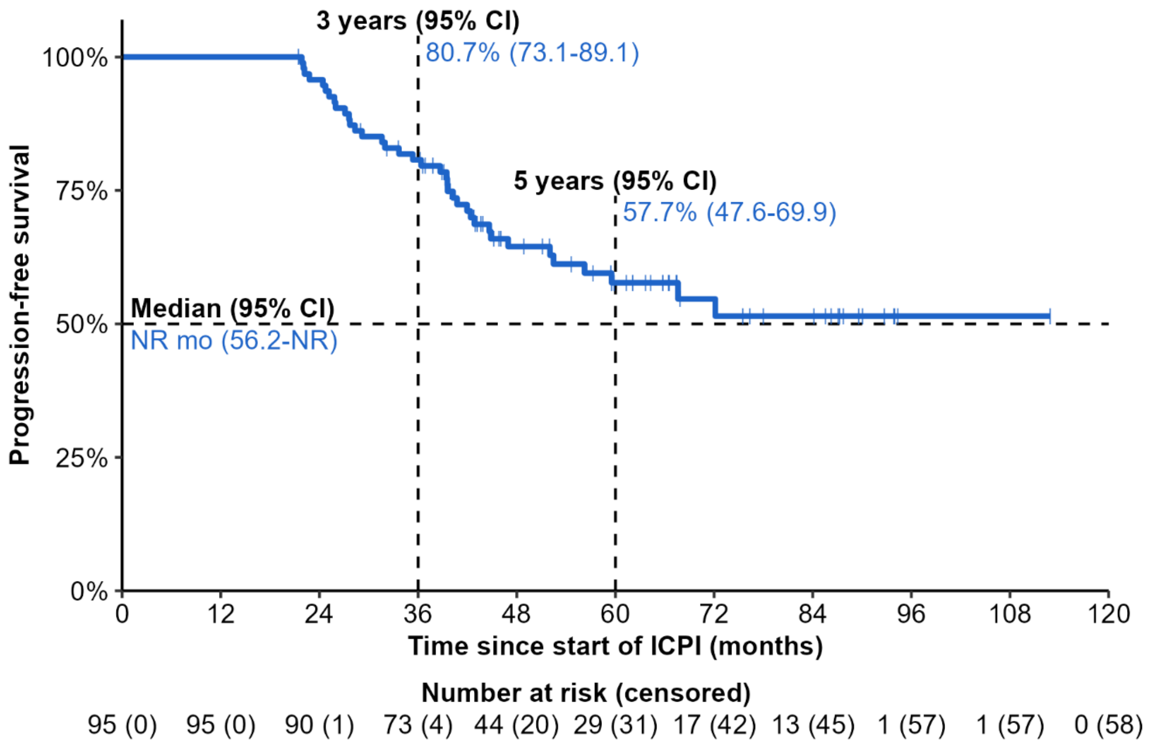

Supplemental Figure 5. Overall survival in the overall population. Abbreviations: CI, confidence interval; ICPI, immune checkpoint inhibitor; mo, month; NR, not reached.

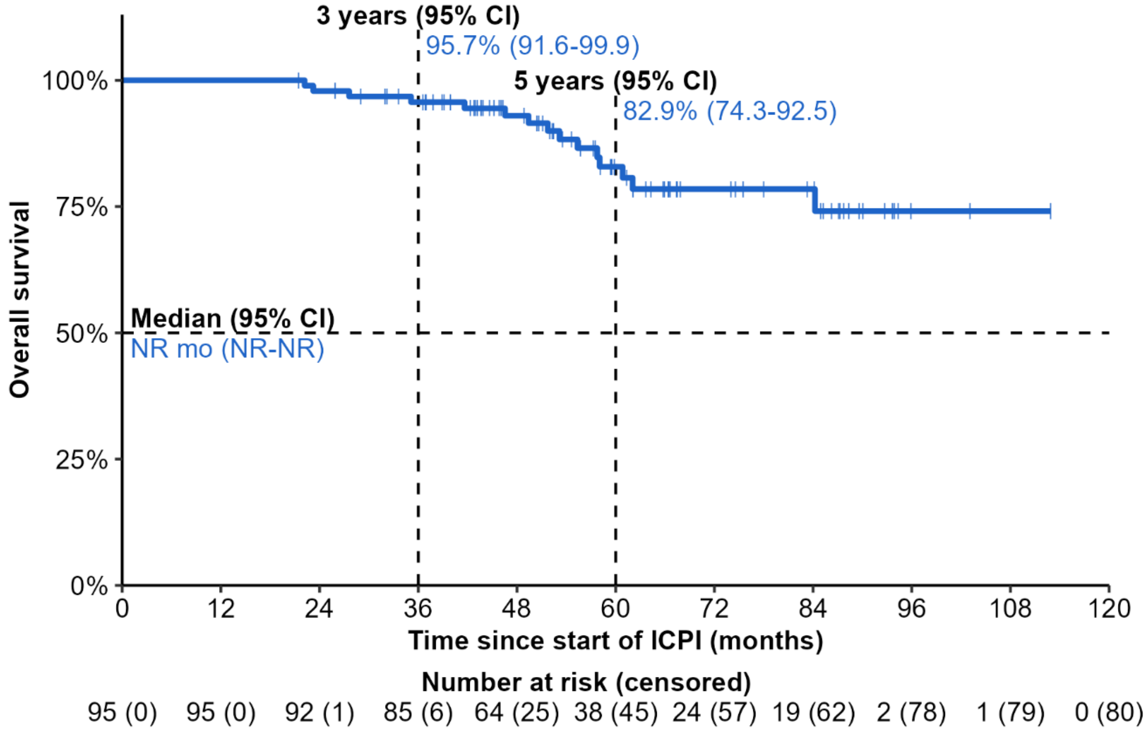

Supplemental Figure 6. Cancer-specific survival in the overall population. Abbreviations: CI, confidence interval; ICPI, immune checkpoint inhibitor; mo, month; NR, not reached.

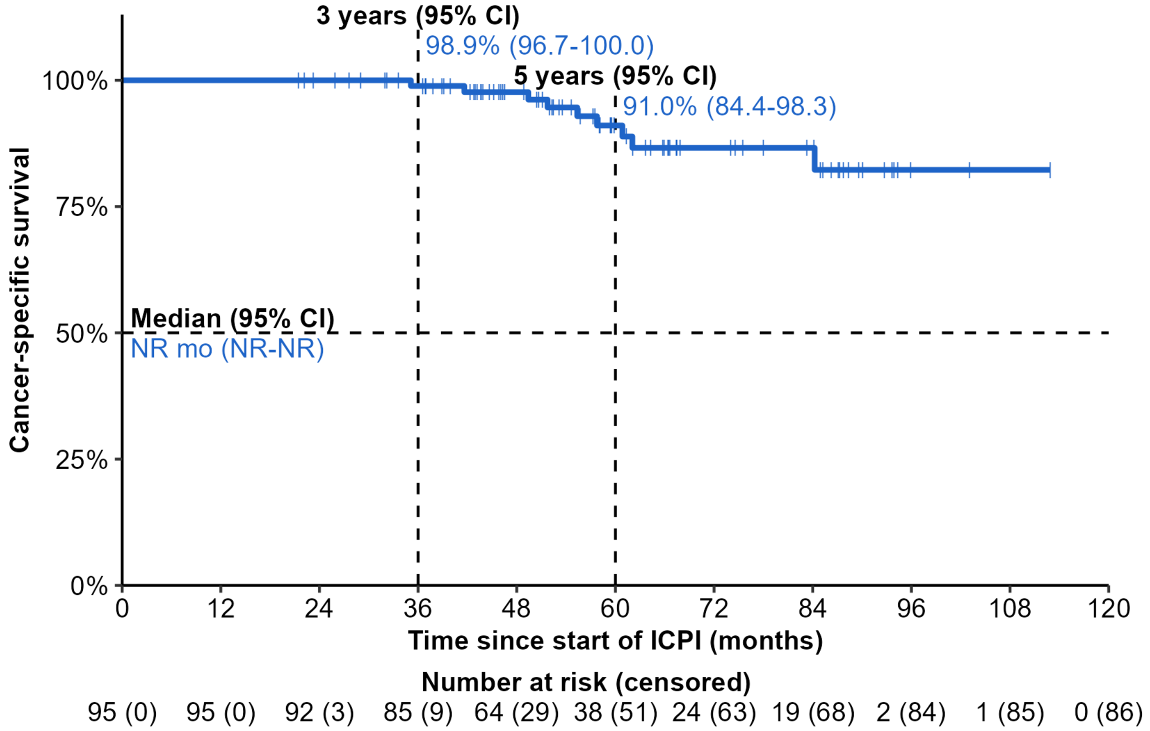

Supplemental Figure 7. Univariate associations between baseline characteristics and progression-free survival, overall survival, and cancer-specific survival. Wald-type 95% confidence intervals are plotted, while log-rank P-values are presented. The hazard ratio for overall survival and cancer-specific survival by best objective response was non-estimable since nobody with complete response experienced death. *Only nephrectomy at baseline is considered here. Abbreviations: ECOG, Eastern Cooperative Oncology Group; ICPI, immune checkpoint inhibitor; IMDC, International Metastatic RCC Database Consortium.

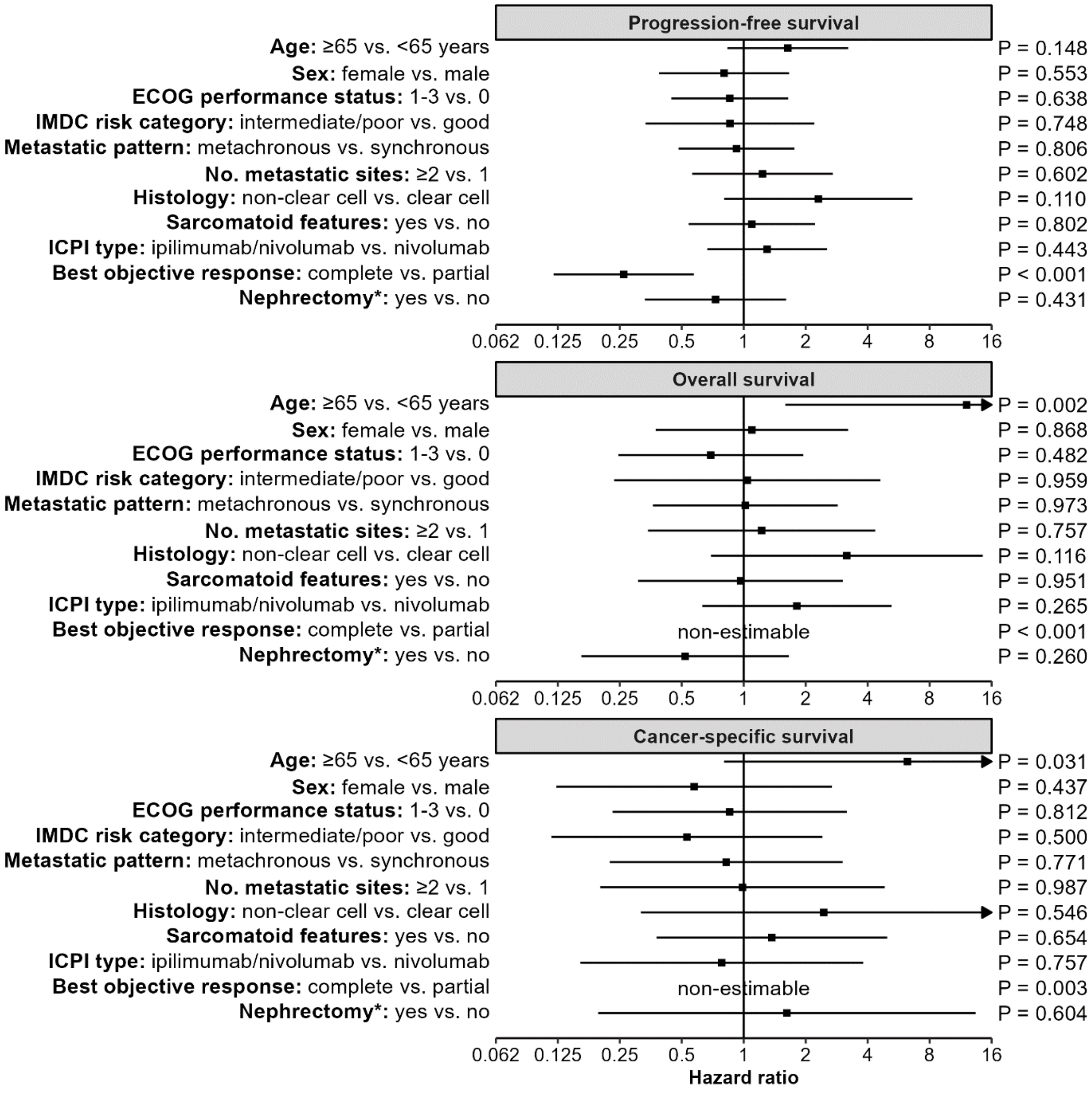

Supplemental Figure 8. Survival functions of progression-free survival, overall survival, and cancer-specific survival by type of ICPI, best objective response, and IMDC risk category. Abbreviations: ICPI, immune checkpoint inhibitor; IMDC, International Metastatic RCC Database Consortium.

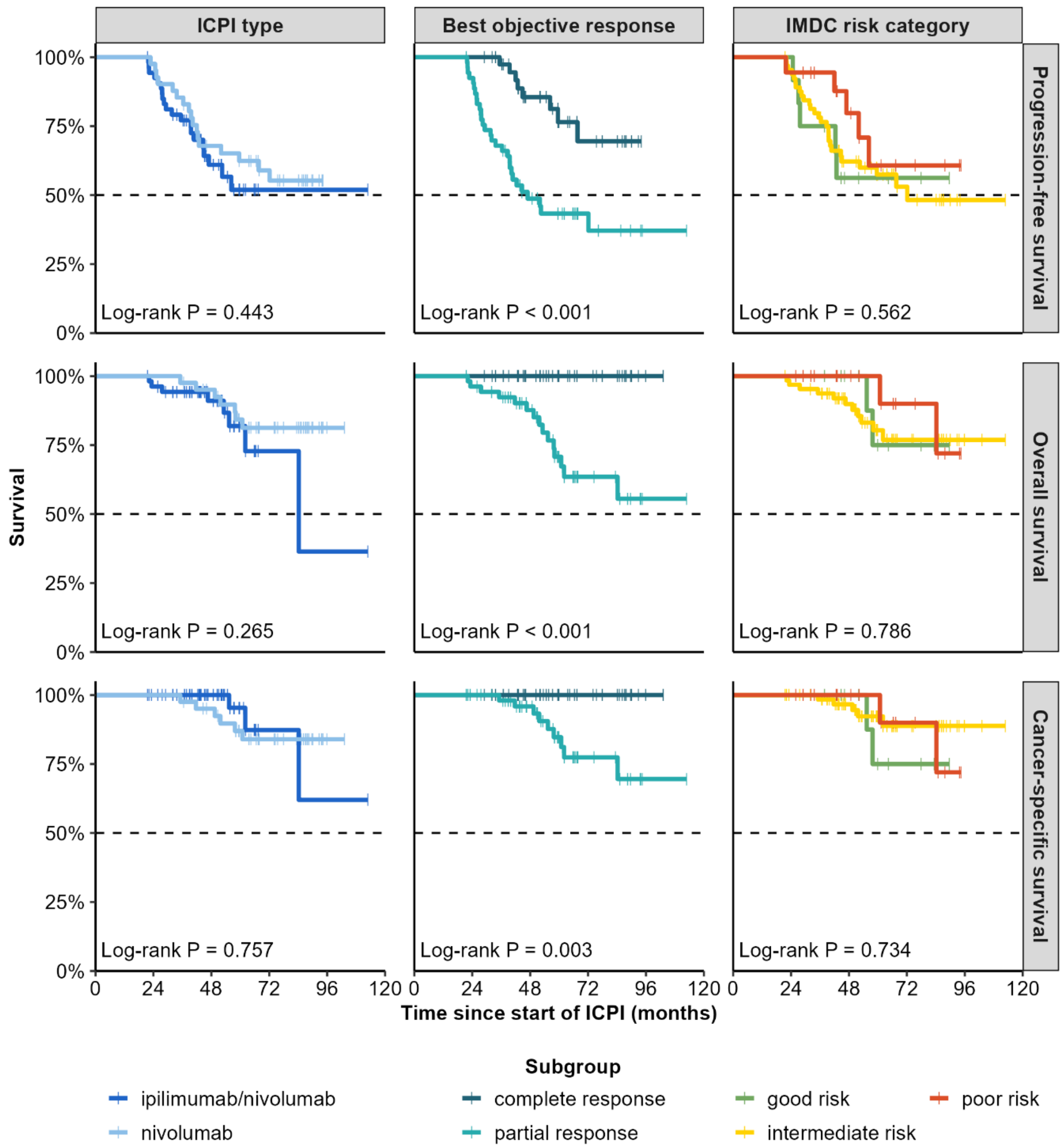

Supplement Figure 9. Cumulative incidence of therapy stop in patients electively discontinuing their ICPI treatment (at any moment). Abbreviations: CI, confidence interval; ICPI immune checkpoint inhibitor; mo, month.

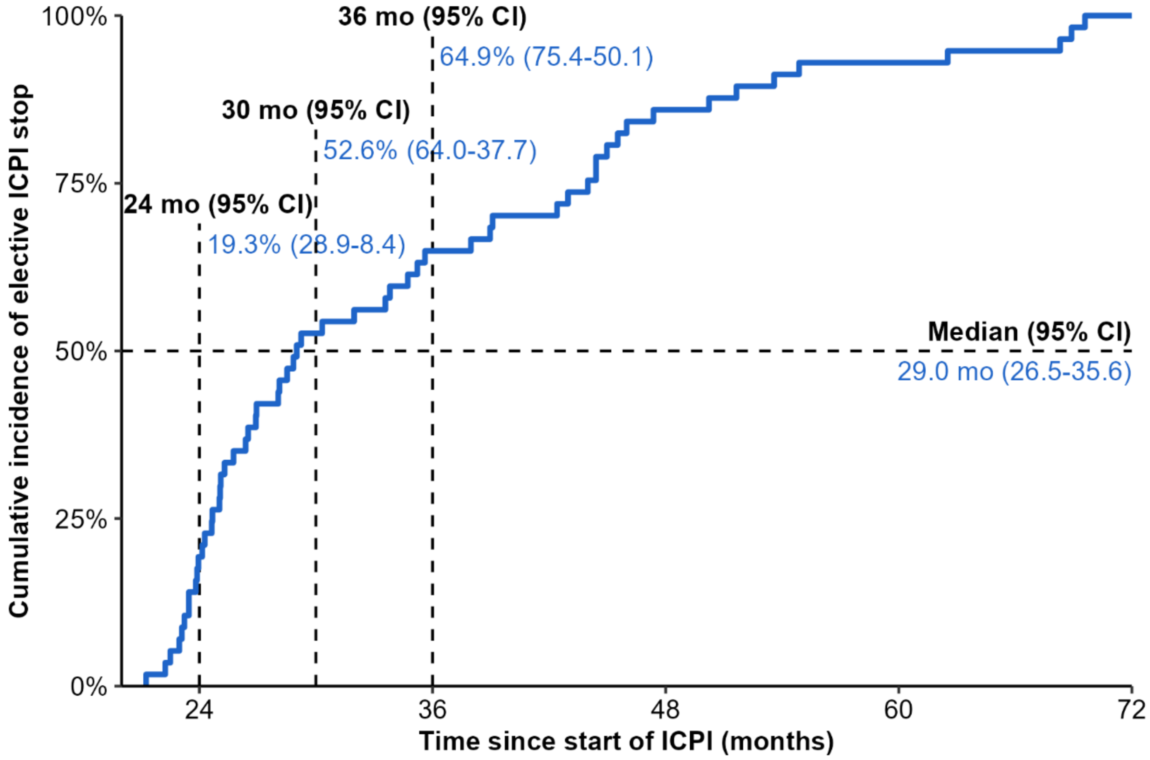

Supplement Figure 10. Survival functions post-discontinuation of progression-free survival, overall survival, and cancer-specific survival in all patients electively discontinuing their ICPI treatment at any moment, by type of ICPI, and by best objective response. Abbreviations: ICPI, immune checkpoint inhibitor.

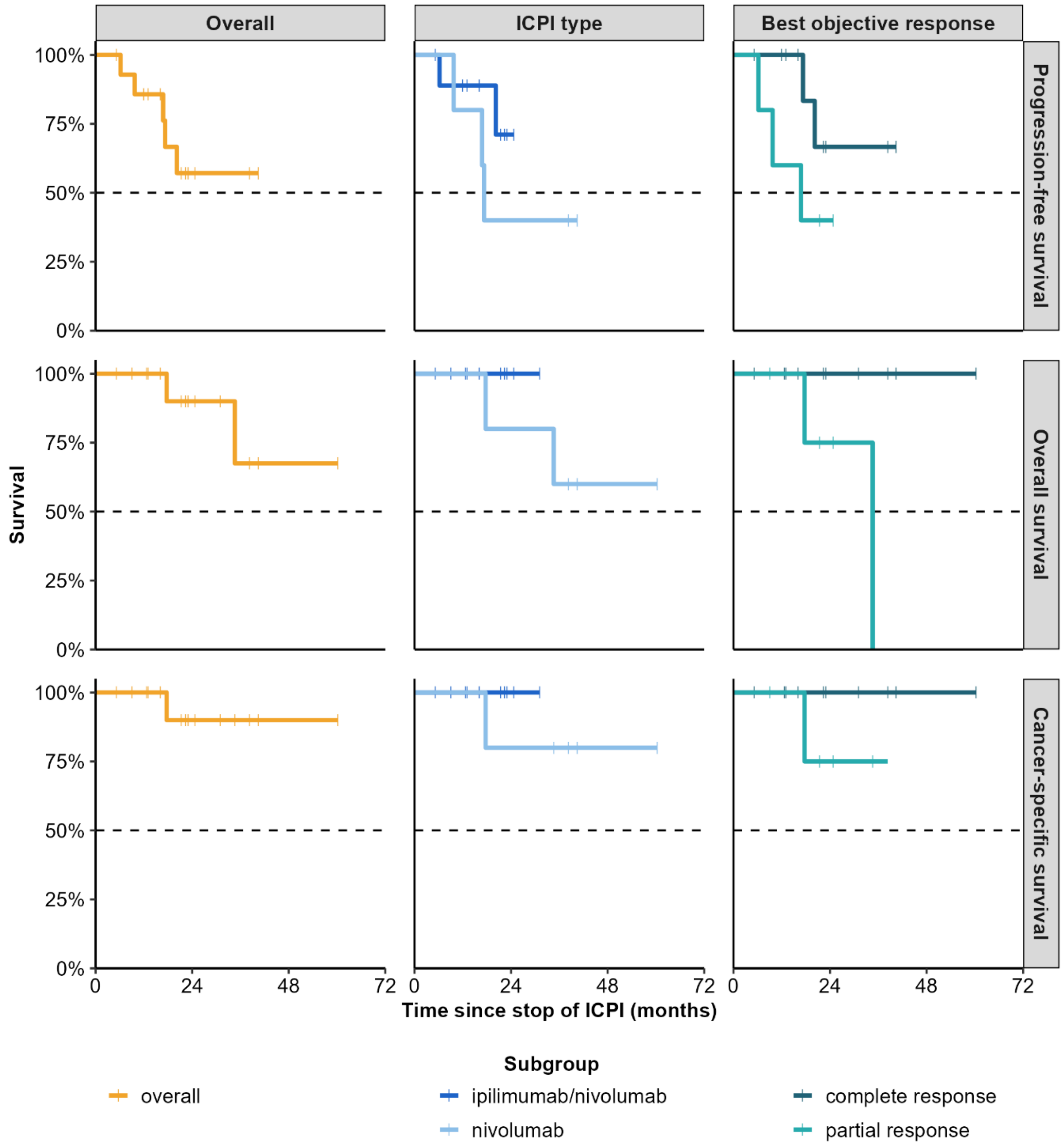

Supplement Figure 11. Forest plot of progression-free survival for ICPI continuation vs. elective ICPI stop between 21-25 months by baseline subgroup. Interaction for IMDC risk category and histology could not be fitted due to sparse data. *Only nephrectomy at baseline is considered here. Abbreviations: ECOG, Eastern Cooperative Oncology Group; HR, hazard ratio; ICPI, immune checkpoint inhibitor; IMDC, International Metastatic RCC Database Consortium.

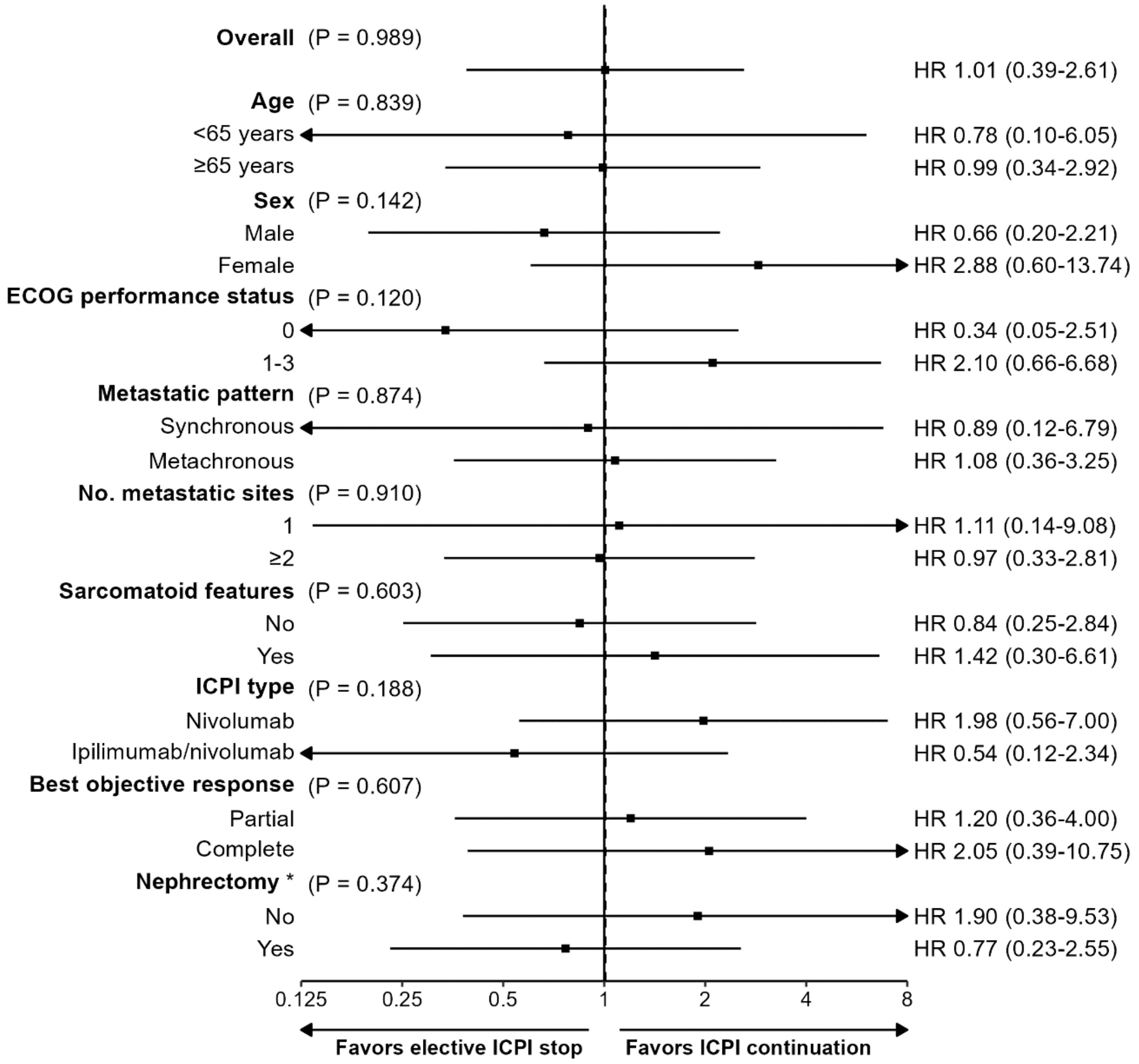

Supplement: Supplementary file 1 [file AO-64-43876-s1.pdf]
